# Internet financial services, environmental technology, and happiness: Implications for sustainable development

**DOI:** 10.1371/journal.pone.0287673

**Published:** 2023-11-08

**Authors:** Lv Yan, Tang Tao, Sana Ullah

**Affiliations:** 1 School of Economics and Management, Hubei University of Education, Wuhan, China; 2 Collaborative Innovation Center for Emissions Trading System Co-constructed by the Province and Ministry, Hubei University of Economics, Wuhan, China; 3 School of Economics, Quaid-i-Azam University, Islamabad, Pakistan; Royal Melbourne Institute of Technology, AUSTRALIA

## Abstract

Integrating the internet and financial services gives people the luxury to reduce financial stress and anxiety by giving consumers more power over their financial situation. Likewise, the adoption of environmental technologies helps improve environmental quality, which positively impacts mental and physical health and thus increases the sense of well-being and happiness. Therefore, the main focus of the study is to analyze the influence of financial services and environmental technologies on happiness. For analyzing the short and long-run impacts of financial services and environmental technologies on happiness, we have utilized the ARDL model and QARDL models. The findings of the ARDL model confirm the positive influence of financial services, environmental technologies, national income, financial development, and education on happiness in the short and long term. Similarly, the QARDL model also suggests the favorable long-run effects of financial services and environmental technologies on happiness at most quantiles. The long-run Wald test confirms the asymmetric influence of all variables on happiness, while in the short-term, excluding education, all other variables exert asymmetric impacts on happiness. Thus, to promote happiness, policymakers should try to increase the role of internet-based financial services and increases investment in research and development activities to enhance environment-related technologies. However, the study is limited to China, it should be expanded to other regions.

## Introduction

In the recent era, with the increasing trend of internet financial services, the Chinese economy is moving towards a more advanced digital financial age. Internet financial services in China consisting of online banking, mobile payment, outstanding financial services, and online loan facilities have not only changed the daily lifestyle of people but also the financial formats [1]. The mobile payment process is closely attached to the daily economic activities of people and has gained great popularity in China [2]. In the mobile payment process, people use smartphones to pay electronic money [3]. Thus, mobile payment unites financial institutions, terminal devices, and the internet to formulate a new setup for making payments [4]. In the present era, China has become the most developed country for making mobile payments around the globe. China ranked at the top for using mobile payments in the world. As approximately 47% population of China is using mobile payments and WeChat Pay and Alipay have almost 810 million mobile payment users. The Chinese economy is gradually converging towards a cashless society. Most prevailing studies considered the impact of internet financial services on the economic behaviors of households [5, 6]. Some researchers have paid attention to the nexus between mobile payment and human well-being and happiness [7, 8]. Only limited literature explored the impact of mobile payments on the happiness of residents.

Given the wide-ranging characteristics of internet financial services and the convenience of mobile payments, we supposed that the residents of China get benefits from the development and popularity of internet financial services [9]. Especially, as a fast and portable payment method, the convenience of internet financial services improves the efficiency of transactions and saves the excessive cost of transactions [10]. Additionally, internet financial services improve the consumption structure of households and enhance the life quality of residents [11]. Moreover, the internet financial services tools in China such as Alipay and WeChat Pay can be used as online social and online lending platforms to accelerate and provides better transformation of information and increase agents for social interactions [12]. In this manner, internet financial services increase the well-being and happiness of residents through various channels. In other words, internet financial services serve as a key basis of production for all kinds of economic and social activity [13]. Hence, internet financial services have enlarged the modification of the globe and delivered remarkable services and welfare that were not noticed in the past.

In this digitalized era, the work style of people has changed immensely. The digitalization and involvement of internet financial services at workplaces have affected the productivity and happiness level of people [14]. Historically, happiness was defined in myriad ways. According to Greek, happiness was defined as a Eudaimonia, which means a good guardian spirit that is required to achieve a valuable life. According to English, happiness is defined through luck and chance. However, in the present time, happiness is defined as a state of mind, the feeling of being satisfied, and glad [15]. Literature provides various measures through which happiness can be determined, such as the happiness index, satisfaction from work, and satisfaction from life [16].

Besides internet financial services, environmental technology is considered another important determinant for raising the living standards of people. Many researchers have shown a positive linkage between environmental innovation and economic growth and stressed further development in environmental technology [17, 18]. Most recent research relates environmental innovation to the quality of life and living standards [19]. Thus, it can be hypothesized that environmental innovation is beneficial for improving living standards by reducing morbidity and enhancing life expectancy, level of education, and happiness. Environmental conditions, social relations, insecurity, and inequality have uncertain associations with environmental technology. Each one of these dimensions needs to be explored separately to obtain the overall effect of environmental innovation on the well-being of residents. From this perspective, happiness is considered an important tool for measuring the impact of environmental innovation on various aspects of quality of life [20]. Environmental innovation is required for the well-being and happiness of residents.

The environment can influence the life quality and happiness level of individuals through various channels. Firstly, environmental condition influences human health both directly (water pollution, air pollution, noise, and hazardous substance) and indirectly (natural disasters and climate change). Secondly, access to a clean environment and clean water enhances the happiness level of residents. Thirdly, people get benefits from environmental amenities that enhance their level of happiness. Lastly, environmental calamities damage the lives and properties of populations. Meanwhile, the environmental condition can be strongly influenced by environmental innovation, both in negative and positive manners. Industrial technology generates waste and pollution, but environmental technology helps in discovering green materials and green sources. Environmental technology is an influential source to foster happiness levels and human well-being. As it is evident from the above discussion the association between environmental technology and happiness is undeniable but this area needs proper empirical investigation [21, 22].

Despite the growing importance of internet financial services and environmental technologies in China, there is a research gap on their impact on happiness. While several studies have examined the relationship between financial development and happiness [23, 24], few have specifically investigated the impact of internet financial services on happiness. Moreover, although there is some research on the relationship between environmental technologies and well-being, little is known about their impact on happiness in the Chinese context. Therefore, there is a need for research that explores how internet financial services and environmental technologies influence happiness in China. This research gap is essential to address, as understanding the relationship between these factors and happiness can inform policies and interventions aimed at promoting sustainable development in China. Previous studies have used outdated estimation techniques [25, 26]. Thus, our research aims to examine the relationship between internet financial services, environmental technology, and happiness in the case of China for the period 1996 to 2020. For empirical investigation, the analysis applied the ARDL and QARDL models. To avoid aggregation biases potentially hidden in aggregate data, our study has conducted a single country-specific analysis for better estimates. There are many examples of studies that have employed ARDL or QARDL methodology to investigate the relationship between variables related to happiness and well-being [24, 27]. Both the ARDL and QARDL models have several advantages, including the ability to estimate long-run relationships between variables even when they are nonstationary, as well as the ability to handle endogeneity and small sample sizes.

This study is motivated by several factors. Firstly, China has experienced rapid economic growth in recent decades, resulting in increased urbanization and industrialization, which have had significant impacts on the environment and people’s well-being [28]. Secondly, the widespread use of internet financial services and the adoption of environmental technologies have emerged as notable trends in China’s economic and social development [29]. Thirdly, internet financial services, such as online banking, mobile payment, and peer-to-peer lending, have become increasingly popular in China, providing convenient and accessible financial solutions to individuals and businesses. These services have the potential to improve financial inclusion, enhance economic opportunities, and increase convenience, which may contribute to people’s happiness by improving their financial well-being and overall life satisfaction. Fourthly, China has been making efforts to address environmental challenges, including air pollution and climate change, through the adoption of various environmental technologies. These technologies have the potential to mitigate environmental degradation, promote sustainable development, and improve people’s living environment, which may have a positive impact on their happiness by enhancing their health, well-being, and quality of life [30].

In recent years, China has experienced rapid economic growth and development, resulting in significant changes in its social, economic, and environmental landscape. Along with this transformation, the adoption of internet financial services and environmental technologies has emerged as notable trends in China’s pursuit of sustainable development. The intersection of internet financial services and environmental technologies raises important questions about their potential impact on people’s well-being and happiness in China. As the pursuit of sustainable development becomes a priority for many countries, including China, understanding the relationship between these factors and happiness is of significant importance. Happiness is a multidimensional concept that encompasses individuals’ subjective well-being, life satisfaction, and overall quality of life. It is considered a key indicator of societal progress and an important goal of sustainable development. The integration of internet financial services and environmental technology can contribute to the achievement of SDG 3 by improving access to healthcare, reducing environmental risks, and promoting sustainable behaviors that support health and well-being [31].

Our research contributes to prevailing literature in the following manners. Firstly, it is a pioneer in examining the nexus between internet financial services and happiness for China and the rest of the world as well. Secondly, we verified and analyzed in-depth mechanisms through which internet financial services promote the happiness level of residents. On the positive side, internet financial services reduce the cost of the transaction, enhance life quality and social interactions, and stimulate human development [32]. Thirdly, this study provides new evidence on the linkage between environmental technology and happiness as the available literature provides inconclusive evidence. Fourthly, our study adopts more advanced estimation techniques, such as ARDL and QARDL, which have the capability to provide long-run and short-run relationships among variables. This is a significant improvement over previous studies, which relied on outdated estimation techniques. By using these advanced approaches, we can obtain a more accurate and comprehensive understanding of the relationship between internet financial services, environmental technologies, and happiness in China. Fifthly, the study can make several theoretical and empirical contributions to the existing literature. Theoretically, the study can contribute to the advancement of the triple bottom line, which is a sustainability framework that considers the economic, social, and environmental impacts of economic activities. By examining how internet financial services and environmental technologies interact and influence happiness in China, the study can further refine this theory and provide empirical evidence on the role of technology in shaping well-being in the context of a developing country like China. Empirically, the study can provide empirical evidence on the impact of internet financial services and environmental technologies on happiness in China. Lastly, this research will help policymakers in such policy formulations that enhance people’s well-being and happiness.

## Theoretical framework, model, and methods

Internet financial services can impact happiness in several ways. One way is by providing access to financial products and services that were previously unavailable to certain segments of the population. This can help to reduce financial stress and anxiety, as well as increase feelings of security and stability [33]. Environmental technology can also impact happiness. One of the most significant ways is by helping to mitigate the negative impacts of climate change, which can improve the quality of life for people and communities around the world. For example, technologies that reduce greenhouse gas emissions can lead to improved air and water quality, which can have a direct impact on physical health and well-being [19]. The United Nations’ Sustainable Development Goals provide a framework for understanding the intersection of internet financial services, environmental technology, and happiness [34]. These goals, which include goals related to sustainable cities and communities, affordable and clean energy, and responsible consumption and production, highlight the interdependence of social, economic, and environmental issues. Achieving these goals requires collaboration between stakeholders from a variety of sectors, including financial services, technology, and environmental conservation. Thus based on the theoretical framework, the model can be written as follows:

$${Happiness}_{t}=\varphi_{0}+\varphi_{1}{DF}_{t}+\varphi_{2}{ET}_{t}+\varphi_{3}GDP+\varphi_{4}{FD}_{t}+\varphi_{5}{EDU}_{t}+\varepsilon_{t}$$ (1)

Specification (1) is the happiness function that relies on digital finance (DF), environmental technology (ET), GDP per capita (GDP), financial development (FD), and education (EDU). We can only get estimates of the long-run coefficients associated with the variables from Eq (1). In this study, our focus is on both the short and long-run estimates. To that end, we must include the short-term mechanics in Eq (1). The first step in doing so is to designate Eq (1) as an error correction structure. In accordance with past studies, we used the bounds-testing method for cointegration and error-correction modeling of Pesaran et al. [35] as indicated by Eq (2):

$${\Delta Happiness}_{t}=\varphi_{0}+\sum_{k=1}^{n}\beta_{1k}{\Delta Happiness}_{t-k}+\sum_{k=0}^{n}\beta_{2k}{\Delta DF}_{t-k}+\sum_{k=0}^{n}\beta_{3k}{\Delta ET}_{t-k}+\sum_{k=0}^{n}\beta_{4k}{\Delta GDP}_{t-k}+\sum_{k=0}^{n}\beta_{5k}{\Delta FD}_{t-k}+\sum_{k=0}^{n}\beta_{6k}{\Delta EDU}_{t-k}+\varphi_{1}{Happiness}_{t-1}+\varphi_{2}{DF}_{t-1}+\varphi_{3}{ET}_{t-1}+\varphi_{4}{GDP}_{t-1}+\varphi_{5}{FD}_{t-1}+\varphi_{6}{EDU}_{t-1}+\varepsilon_{t}$$ (2)

Specification (2) is formally known as the ARDL model. One of the biggest contributions of this method to econometric literature is its strength to provide short and long-run estimates. Coefficient estimates associated with Δ variables represent short-run impacts, whereas estimates of φ_2_ and φ_6_ divided by -φ_1_ indicate long-run impacts under the aforementioned specification (2). Pesaran et al. [35] suggest a test for cointegration that should be used if these long-run estimates are to have any useful value. To determine the significance of ECM, which needs to be negative, as suggested by Pesaran et al. [35]. They show that these tests’ dispersion is nonstandard; thus, they develop new critical values that consider the variables’ integrating characteristics. In fact, using this method, variables may be a combination of I(0) and I (1). The pre-unit-root analysis is unnecessary because many macro variables have these characteristics. Another important benefit of this method is its power to perform well in cases where data length is small across time. Lastly, the issues of endogeneity and heterogeneity can be addressed by applying this method because it includes a short-run adjustment mechanism in the model.

A few other diagnoses are also presented to check whether our estimates are valid or not. First, the Lagrange Multiplier (LM) measure is used to prove whether the residuals are autocorrelation free or not. Ramsey’s RESET test is applied to see whether there is any specification bias in the model. Both these tests have Chi-square (χ2) distribution with one degree of freedom. We execute CUSUM and CUSUM-sq checks on the residuals of the framework to determine the robustness of short-run and long-run estimations. Although the baseline model is ARDL; however, we have also used the QARDL to check the robustness of our results. The QARDL model provides correct estimates when non-normality occurs.

### Data and descriptive analysis

The present study aims to explore the impact of internet financial services and environmental technology on happiness in the case of China. The study collects time-series data for the period 1998–2021 and the definitions and descriptive statistics related to data series are given in Table 1. Happiness measures the subjective well-being or life satisfaction of individuals in a given population. It is measured through a happiness index ranging from 0 to 10, where 0 means least happy and 10 means most happy. The happiness index is measured using a range of variables that are believed to contribute to happiness, including social support, economic prosperity, freedom, health, and perceptions of corruption and generosity. Internet financial services in literature are measured through two proxy variables. These are ATM (per 100,000 adults) and Debit card (% age 15 and above). Ahmed et al. [36] reveal that environmental technology measures the level of technology and innovation that is being used to address environmental problems and promote sustainable development. It is measured as environment-related technologies in terms of total patents. Our study included GDP per capita, financial development, and education as control variables in the model. GDP per capita is a measure of a country’s economic output per person. It is taken as constant at 2015 US$. Li et al. [37] described that financial development refers to the level of development of a country’s financial sector. It is measured through the financial development index. Financial development is a comprehensive measure that assesses and ranks countries based on the depth, access, and efficiency of their financial institutions and markets. The computation of the aggregate measure involves the incorporation of both the financial institution index and the financial market index. Ullah et al. [38] state that education refers to the level of formal education completed by an individual, as well as the level of education available within a given population or society. In our study, the average year of schooling (age 15+) is used as a proxy measure of education. The required data series are collected from the IMF and the World Bank.

**Table 1:** Definitions and descriptive statistics.

Variables	Definitions	Mean	Median	Max	Min	Std. Dev.	Skewness	Kurtosis	Jarque-Bera	Prob.
Happiness	Happiness index ranges from 0 (least happy) to 10 (most happy)	4.630	4.660	5.706	3.476	0.557	-0.158	1.912	5.345	0.069
ATM	ATMs per 100,000 adults	2.983	2.981	4.549	0.839	1.189	-0.077	1.566	8.666	0.013
Debit	Debit card (% age 15+)	3.485	3.588	4.339	2.215	0.633	-0.469	2.166	6.572	0.037
ET	Environment-related technologies (total patents)	10.33	10.47	12.05	8.160	1.320	-0.248	1.676	8.329	0.016
GDP	GDP per capita (constant 2015 US$)	8.469	8.542	9.364	7.464	0.591	-0.195	1.656	8.161	0.017
FD	Financial development index	0.746	0.752	0.793	0.662	0.036	-0.704	2.607	8.910	0.012
EDU	Average years of total schooling, age 15+, total	7.415	7.466	8.109	6.483	0.404	-0.450	2.348	5.144	0.076

Descriptive statistics provides results for various data-based tests. The mean values for variables are reported as: 4.630 for happiness, 2.983 for ATM, 3.485 for Debit, 10.33 for ET, 8.469 for GDP, 0.746 for FD, and 7.415 for EDU. The standard deviations are reported as: 0.557 for happiness, 1.189 for ATM, 0.633 for Debit, 1.320 for ET, 0.591 for GDP, 0.036 for FD, and 0.404 for EDU. J-B statistics reveal that none of the data series is normally distributed in our model.

## Results and discussion

Before regressing a model, it is important to test for stationarity in the data. This is because if the data is not stationary, the estimated coefficients of the regression model may be biased or inconsistent. There are several tests that can be used to check for stationarity in time series data, but we have used the ADF test, PP test, and DF-GLS test. These tests can help to determine whether the time series is stationary or non-stationary and can provide insights into the underlying dynamics of the series. The outcome of these three tests is given in Table 2. According to ADF and PP tests, DEBIT and EDU are found stationary at the level and the remaining series are found stationary at first difference. According to the DF-GLS test, FD and EDU are stationary at the level and the remaining series becomes stationary at first difference. Hence, the mixture of I(0) and I(1) integrated series validate the use of ARDL and QARDL approaches for regression purposes.

**Table 2:** Unit root results.

	ADF		PP		DF-GLS	
	I(0)	I(1)	I(0)	I(1)	I(0)	I(1)
Happiness	1.425	-4.365***	-0.758	-2.758*	2.012	-1.687*
ATM	-0.825	-2.688*	-1.925	-3.658***	0.895	-1.678*
DEBIT	-4.325***		-4.021***		-0.165	-1.657*
ET	-2.125	-2.879*	-1.542	-2.987**	-0.169	-1.721*
GDP	-0.778	-2.689*	-1.621	-2.754*	0.821	-2.587***
FD	-1.987	-4.987***	-1.754	-5.325***	-2.754***	
EDU	-2.788*		-3.542***		-2.354**	

**Note:** *** p<0.01

** p<0.05

* p<0.1

The long-run dynamics and short-run dynamics under the ARDL model are given in Table 3. In model 1, the coefficient estimate of the ATM is 0.434. This means that a 1% increase in the ATM is associated with a 0.434% increase in happiness. It suggests that over time, a sustained increase in the ATM is likely to lead to a corresponding increase in happiness. In support of our finding, Meng & Xiao [33] reports that internet financial services can provide access to financial services to individuals who may have been excluded from traditional financial systems due to factors such as distance, cost, or lack of documentation. Increased financial inclusion can lead to greater financial stability and security, which can contribute to happiness. Rahman et al. [13] described that internet financial services can also provide individuals with greater control over their finances. For example, mobile banking apps and budgeting tools can help people track their spending and make informed financial decisions. This can reduce financial stress and anxiety, which can contribute to increased happiness. In a similar vein, Zhao et al. [8] claimed that internet financial services can make it easier and more convenient to access financial services. For example, mobile payments and online banking can save time and effort compared to traditional in-person transactions. This can reduce stress and increase feelings of control and autonomy, which are important factors in happiness.

**Table 3:** Results of ARDL.

	(1)				(2)			
Variable	Coeff.	Std. Error	t-Stat	Prob.	Coeff.	Std. Error	t-Stat	Prob.
**Long-run**								
ATM	0.434**	0.220	1.975	0.052				
DEBIT					1.447***	0.387	3.736	0.000
ET	1.077**	0.516	2.086	0.040	1.249***	0.251	4.968	0.000
GDP	2.945*	1.633	1.803	0.075	1.769***	0.515	3.434	0.001
FD	0.247***	0.084	2.937	0.004	0.386**	0.184	2.094	0.039
EDU	1.031**	0.450	2.291	0.025	2.240***	0.516	4.342	0.000
**Short-run**								
ATM	0.191***	0.040	4.796	0.000				
ATM(-1)	0.342***	0.070	4.873	0.000				
ATM(-2)	0.150***	0.041	3.639	0.001				
DEBIT					0.596***	0.166	3.591	0.001
DEBIT(-1)					0.982***	0.299	3.283	0.002
ET	0.265***	0.049	5.374	0.000	0.304***	0.052	5.879	0.000
ET(-1)	0.494***	0.092	5.355	0.000	0.547***	0.094	5.828	0.000
ET(-2)	0.205***	0.057	3.626	0.001	0.217***	0.064	3.410	0.001
GDP	0.074**	0.030	2.415	0.018	0.761***	0.230	3.315	0.001
FD	0.193*	0.104	1.864	0.066	1.369***	0.411	3.332	0.001
FD(-1)	0.361**	0.155	2.336	0.022	0.544**	0.227	2.392	0.019
FD(-2)	0.180*	0.094	1.904	0.060	0.032	0.047	0.679	0.499
EDU	0.353*	0.194	1.820	0.072	0.387*	0.201	1.925	0.058
EDU(-1)	0.311*	0.184	1.693	0.094	0.340*	0.190	1.794	0.076
C	11.48*	6.854	1.676	0.097	5.211***	1.808	2.882	0.005
**Diagnostics**								
F-test	18.25***				16.22			
ECM(-1)*	-0.378***	0.077	-4.926	0.000	-0.314***	0.091	-3.446	0.001
LM	1.023				1.321			
RESET	2.021				0.875			
CUSUM	S				S			
CUSUM-sq	S				S			

**Note:** *** p<0.01

** p<0.05

* p<0.1

The coefficient estimate of environmental technology is 1.077. This shows that a 1% increase in the use or availability of environmental technology is associated with a 1.077% increase in happiness. This suggests that in the long-run, using or having access to environmental technology is likely to have a positive impact on happiness. Our findings are supported by various studies. For example, Aldieri et al. [19] described that environmental technology can help to reduce pollution and mitigate the impacts of climate change, leading to improved environmental conditions. Exposure to cleaner air, water, and surroundings can have a positive effect on mental and physical health, which can contribute to increased happiness. Findings infer that environmental technologies, such as smart thermostats and energy-efficient appliances, can make it easier and more convenient for people to adopt sustainable behaviors. This can reduce stress and increase feelings of control and autonomy, which are important factors in happiness. In support, Brulé & Munier [39] reveals that environmental technologies can also facilitate social connections, which are a key component of happiness. For example, community gardens and urban agriculture projects can bring people together around a shared goal, and renewable energy cooperatives can create opportunities for collective action and engagement.

The coefficient estimate of GDP per capita is 2.945. It reveals that a 1% upsurge in GDP per capita is associated with a 2.945% upsurge in happiness. This suggests that in the long-run, higher GDP per capita is likely to be associated with higher levels of happiness. The coefficient estimate of financial development is 0.247. It infers that a 1% increase in financial development is associated with a 0.247% increase in happiness. Li et al. [37] revealed that financial development refers to the development of financial markets, institutions, and services, such as banks, stock markets, and insurance companies. This result suggests that in the long run, an increase in financial development is likely to lead to an increase in happiness. The coefficient estimate of education is 1.031. It reveals that a 1% upsurge in education is associated with a 1.031% upsurge in happiness. This suggests that in the long-run, higher education is likely to be associated with higher levels of happiness. In model 2, the coefficient estimate of DEBIT is 1.447. This means that a 1% increase in DEBIT is associated with a 1.447% increase in happiness. This suggests that, in the long-run, using debit more frequently or having a higher level of debit usage is likely to lead to an increase in happiness. In model 2, long-run coefficient estimates of other variables i.e. ET, GDP, FD, and EDU are found consistent.

In the short-run, the coefficient estimates of ATM, DEBIT, and ET are 0.191, 0.596, and 0.265, respectively. This means that a 1% increase in ATM, DEBIT, and ET is associated with a 0.191%, 0.596%, 0.265% increase in happiness in the short-run. This suggests that in the short-run, the use or availability of ATMs, the use of debit cards, and the availability of environmental technology are likely to have positive effects on happiness.

Several diagnostic tests have been used to assess the quality and reliability of the ARDL model results. For instance, the F-test is used to test the overall significance of the ARDL model. The ECM is used to test for the existence of a long-run relationship between the variables in the ARDL model. Both the F-test and ECM test provide significant results. The LM test is used to test for the presence of serial correlation in the residuals of the ARDL model and no evidence of serial correlation is detected in our models. Ramsey RESET test confirms the correct specification of both models. The CUSUM test and CUSUM-squared test are confirming the stability of both models. The QARDL approach is used to capture the abnormal distribution and to assess the robustness of the relationship between the variables. Table 4 reports the results for the QARDL model. The impact of ATM on happiness is found significant and positive at all quantiles except 0.05, revealing that an upsurge in the use of ATM tends to enhance happiness in the long-run. The relationship between Debit and happiness is found significant and positive at all quantiles in the long run (see Table 5). The coefficient estimates of environmental technology are found significant and positive at quantiles 0.70 to 0.95, describing that ET tends to enhance happiness at these quantiles in the long run. The Wald test results are reported in Table 6.

**Table 4:** Results of QARDL.

			Long-run					Short-run						
	ECM	C	ATM	ET	GDP	FD	EDU	ATM	ET	ET(-1)	GDP	GDP(-1)	FD	EDU
0.05	-0.423***	1.779	0.131	0.069	0.105	0.118	0.813***	0.144	0.166	0.124	1.043	1.007	0.018	0.109
	(-7.920)	(0.726)	(1.377)	(0.316)	(0.225)	(0.173)	(5.656)	(1.243)	(1.418)	(1.481)	(1.395)	(1.345)	(0.153)	(0.263)
0.10	-0.371***	1.793	0.129**	0.063	0.053	0.080	0.653***	0.118	0.164	0.133	0.629	0.554	0.011	0.178
	(-6.799)	(1.255)	(2.189)	(0.437)	(0.166)	(0.137)	(5.545)	(0.832)	(1.115)	(1.375)	(1.277)	(1.317)	(0.080)	(0.430)
0.20	-0.375***	2.419***	0.139***	0.018	0.291	0.047	0.589***	0.063	0.163	0.146	0.715	0.640	0.102	0.020
	(-6.026)	(2.904)	(4.909)	(0.208)	(1.629)	(0.222)	(7.502)	(0.699)	(0.852)	(1.270)	(1.216)	(1.280)	(0.578)	(0.035)
0.30	-0.364***	2.443***	0.142***	0.027	0.333*	0.132	0.566***	0.062	0.130	0.111	0.835	0.760	0.080	0.032
	(-3.027)	(3.002)	(6.187)	(0.333)	(1.907)	(0.788)	(8.064)	(0.650)	(0.331)	(1.029)	(1.082)	(1.278)	(0.439)	(0.055)
0.40	-0.159***	2.618***	0.141***	0.049	0.393**	0.179	0.556***	0.025	0.024	0.025	0.174	0.125	0.009	0.188
	(-2.972)	(2.710)	(6.589)	(0.508)	(2.007)	(1.124)	(7.217)	(0.293)	(0.535)	(0.375)	(0.756)	(0.576)	(0.117)	(0.440)
0.50	-0.153**	2.628***	0.122***	0.035	0.433***	0.459*	0.529***	0.063	0.016	0.016	0.186	0.133	0.033	0.770
	(-2.420)	(3.128)	(5.120)	(0.418)	(2.831)	(1.956)	(6.539)	(0.397)	(0.135)	(0.095)	(0.468)	(0.345)	(0.257)	(0.338)
0.60	-0.069**	2.435***	0.118***	0.015	0.435***	0.652***	0.495***	0.137	0.022	0.010	0.024	0.033	0.053	0.925
	(-2.121)	(3.763)	(5.704)	(0.938)	(3.343)	(2.658)	(8.383)	(1.400)	(0.181)	(0.079)	(0.052)	(0.071)	(0.627)	(1.147)
0.70	-0.135**	2.465***	0.107***	0.011*	0.490***	0.895***	0.461***	0.244***	0.041	0.029	0.145	0.233	0.071	0.761
	(-2.005)	(3.678)	(5.411)	(1.802)	(2.961)	(4.016)	(10.97)	(2.884)	(0.447)	(0.258)	(0.344)	(0.594)	(0.947)	(1.549)
0.80	-0.133*	2.837***	0.109***	0.071**	0.668***	1.184***	0.422***	0.227***	0.040	0.018	0.002	0.097	0.121*	0.888*
	(-1.746)	(4.370)	(5.570)	(2.084)	(3.921)	(6.746)	(13.56)	(3.032)	(0.395)	(0.135)	(0.006)	(0.246)	(1.747)	(1.871)
0.90	-0.177**	3.941***	0.151***	0.232***	0.928***	0.824***	0.448***	0.188***	0.088	0.069	0.019	0.053	0.181*	0.901**
	(-2.053)	(6.843)	(2.809)	(3.216)	(6.470)	(2.607)	(12.23)	(3.607)	(1.074)	(0.590)	(0.055)	(0.166)	(1.901)	(2.117)
0.95	-0.256**	4.474***	0.155***	0.292***	1.066***	0.798***	0.443***	0.224***	0.114	0.085	0.043	0.054	0.197**	0.534**
	(-2.286)	(10.32)	(3.512)	(6.550)	(9.921)	(2.900)	(12.22)	(5.061)	(1.414)	(1.015)	(0.158)	(0.209)	(2.328)	(2.431)

**Note:** *** p<0.01

** p<0.05

* p<0.1

**Table 5:** Results of QARDL-robustness.

			Long-run					Short-run						
	ECM	C	Debit	ET	GDP	FD	EDU	Debit	ET	ET(-1)	GDP	GDP(-1)	FD	EDU
0.05	-0.434***	10.55***	1.507***	0.568***	0.369	0.488	2.331***	1.022***	0.151**	0.263	0.120	0.167	0.214	1.081
	(-6.244)	(3.631)	(5.765)	(4.106)	(1.221)	(0.950)	(7.060)	(4.141)	(2.348)	(0.741)	(0.202)	(0.281)	(1.185)	(1.492)
0.10	-0.346***	9.985***	1.454***	0.575***	0.366	0.269	2.238***	1.015***	0.209***	0.284	0.583	0.392	0.085	1.074
	(-3.868)	(3.007)	(4.895)	(3.871)	(1.117)	(0.446)	(5.945)	(3.748)	(2.866)	(0.309)	(0.995)	(0.590)	(0.406)	(1.455)
0.20	-0.196***	8.913***	1.120**	0.396*	0.025	0.107	1.837**	1.395***	0.217**	0.330	0.620	0.835	0.050	0.968
	(-3.002)	(2.580)	(2.252)	(1.916)	(0.039)	(0.166)	(2.473)	(4.910)	(2.110)	(0.006)	(0.606)	(0.819)	(0.335)	(1.422)
0.30	-0.205***	8.041***	0.839***	0.246**	0.256	0.338	1.499***	1.427***	0.275**	0.391	0.539	0.793	0.132	0.791
	(-4.510)	(3.512)	(3.125)	(2.336)	(1.167)	(0.855)	(4.195)	(6.001)	(2.443)	(0.248)	(0.625)	(0.935)	(1.227)	(1.321)
0.40	-0.183***	7.206***	0.730***	0.249***	0.200	0.450	1.407***	1.536***	0.324***	0.450	0.708	0.964	0.121	0.617
	(-4.860)	(4.361)	(3.453)	(2.777)	(1.092)	(1.554)	(4.756)	(6.681)	(3.205)	(0.249)	(0.871)	(1.225)	(1.547)	(1.319)
0.50	-0.185***	7.640***	0.850***	0.310***	0.098	0.390	1.549***	1.507***	0.327**	0.455	0.553	0.822	0.087	0.546
	(-3.856)	(3.809)	(3.230)	(3.199)	(0.499)	(1.054)	(4.196)	(4.589)	(2.520)	(0.508)	(0.467)	(0.731)	(0.963)	(1.271)
0.60	-0.200***	7.404***	0.797**	0.293***	0.168	0.503	1.448***	1.364***	0.270**	0.416	0.061	0.389	0.132*	0.373
	(-4.488)	(3.295)	(2.464)	(2.663)	(1.273)	(1.510)	(3.263)	(3.634)	(2.197)	(0.721)	(0.052)	(0.356)	(1.795)	(1.222)
0.70	-0.209***	8.111***	0.946**	0.372**	0.191*	0.327**	1.646**	1.220***	0.212***	0.363	0.136	0.203	0.132***	0.257
	(-7.325)	(2.701)	(2.250)	(2.154)	(1.879)	(2.155)	(2.212)	(6.421)	(2.724)	(0.881)	(0.497)	(0.788)	(2.761)	(1.564)
0.80	-0.216***	10.98***	1.467***	0.537***	0.253**	0.142**	2.327***	1.241***	0.239***	0.392	0.088	0.257	0.158***	0.378**
	(-5.044)	(7.166)	(3.933)	(3.070)	(2.227)	(2.326)	(4.751)	(5.008)	(3.368)	(1.014)	(0.291)	(0.895)	(3.575)	(1.971)
0.90	-0.297***	10.85***	1.457***	0.548***	0.292***	0.184**	2.308***	1.338***	0.221***	0.398	0.348	0.722	0.173***	0.052**
	(-9.153)	(7.356)	(3.955)	(3.133)	(2.582)	(2.444)	(4.782)	(9.184)	(3.969)	(1.262)	(1.076)	(1.131)	(2.863)	(2.261)
0.95	-0.259***	10.88***	1.456***	0.545***	0.350***	0.233***	2.299***	1.212***	0.227***	0.424	0.300	0.712	0.211***	0.172**
	(-9.313)	(9.470)	(5.121)	(4.023)	(2.568)	(2.691)	(6.199)	(11.01)	(4.197)	(1.398)	(1.512)	(1.422)	(2.724)	(2.398)

**Note:** *** p<0.01

** p<0.05

* p<0.1

**Table 6:** Results of the Wald test.

	F-statistics	Prob.	F-statistics	Prob.
**Long-run**				
ATM	5.654***	0.000		
DEBIT			6.658***	0.000
ET	6.658***	0.000	6.001***	0.000
GDP	7.891***	0.000	5.325***	0.000
FD	12.02***	0.000	10.02***	0.000
EDU	12.33***	0.000	8.555***	0.000
**Short-run**				
ATM	4.021***	0.002		
DEBIT			5.658***	0.000
ET	0.123	0.935	0.275	0.281
ET(-1)	0.007	0.984	0.222	0.384
GDP	5.365***	0.000	3.927**	0.012
GDP(-1)	0.686	0.395	0.765	0.726
FD	5.661***	0.000	5.321***	0.001
EDU	4.988***	0.000	3.912**	0.021
ECM	8.025***	0.000	7.324***	0.003

**Note:** *** p<0.01

** p<0.05

* p<0.1

## Conclusion and implications

China has experienced significant economic growth and urbanization in recent decades, resulting in various challenges related to sustainable development, including environmental degradation, resource depletion, and social inequalities. In response to these challenges, the Chinese government has been actively promoting the adoption of internet financial services and environmental technologies as part of its sustainable development strategies. The impact of internet financial services and environmental technologies on happiness, as a multidimensional measure of well-being, has gained attention in recent research. Happiness is not only an essential aspect of individual well-being but also a societal indicator of progress and development. Studies have shown that access to financial services and environmental sustainability are closely related to individuals’ happiness, as they can improve economic opportunities, environmental quality, and social welfare. However, there is limited research that has specifically examined the impact of internet financial services and environmental technologies on happiness in China, and their implications for sustainable development. Therefore, this study aims to fill this research gap by investigating the impact of internet financial services and environmental technologies on happiness by applying ARDL and QARDL models.

For analyzing the short and long-run impacts of ATM, DEBIT, ET, GDP, FD, and EDU, on happiness, we have utilized the ARDL model and QARDL models. The findings of the ARDL model confirm the positive influence of ATM, DEBIT, ET, GDP, FD, and EDU on happiness in the short and long term. Similarly, the QARDL model also suggests the favorable long-run effects of ATM, DEBIT, ET, and EDU on happiness at most quantiles, while the GDP and FD positively impact happiness only at higher quantiles. In the short term, the estimates of the QARDL model are insignificant at most quantiles for most of the variables. The long-run Wald test confirms the asymmetric influence of ATM, DEBIT, ET, GDP, FD, and EDU on happiness, while in the short-term, excluding EDU, all other variables exert asymmetric impacts on happiness.

Following are some practical policy options for experts. The relationship between financial services and happiness is positive. The authorities should improve financial service provision, especially for low-income people and those living in remote locations. This might entail taking steps like opening additional financial institution facilities in neglected regions or rewarding fintech businesses to create unique options for individuals lacking access to conventional banking services. The authorities should try to teach individuals how to handle their funds efficiently via financial literacy workshops. This may make people more aware of their credit, investment, and borrowing alternatives, which might result in more stable finances and general pleasure. Moreover, our findings suggest that increased environment-related technologies increase happiness; hence, the authorities should spend money on environmental technology research and development to hasten their adoption and lower their prices. This could result in the creation of novel, creative options that are more beneficial and effective in minimizing environmental damage, which might enhance pleasure.

This study has several limitations that should be acknowledged. The study’s findings are specific to the Chinese context and may not be directly generalizable to other countries with different socio-economic, cultural, or institutional contexts. While this research is currently examining the symmetric association among selected variables, however, the asymmetric impact of internet financial services and environmental technology on happiness can be explored in future analysis. Future research should also conduct cross-country comparisons to explore the similarities and differences in the impact of internet financial services, environmental technologies, and happiness across different countries. This could provide insights into the contextual factors that influence these relationships and help in identifying best practices.

## Supporting information

S1 Dataset (XLSX)
